# Immunomodulatory and Metabolic Changes after Gnetin-C Supplementation in Humans

**DOI:** 10.3390/nu11061403

**Published:** 2019-06-21

**Authors:** Yuya Nakagami, Susumu Suzuki, J. Luis Espinoza, Lam Vu Quang, Megumi Enomoto, Souichi Takasugi, Ayano Nakamura, Takayuki Nakayama, Hiroya Tani, Ichiro Hanamura, Akiyoshi Takami

**Affiliations:** 1 Department of Internal Medicine, Division of Hematology, Aichi Medical University School of Medicine, Nagakute, Aichi 480-1195, Japan; nakagami.yuuya.223@mail.aichi-med-u.ac.jp (Y.N.); quanglamvu1991@gmail.com (L.V.Q.); takasugi.souichi.387@mail.aichi-med-u.ac.jp (S.T.); ayano.n@aichi-med-u.ac.jp (A.N.); tnaka@aichi-med-u.ac.jp (T.N.); hanamura@aichi-med-u.ac.jp (I.H.); 2 Department of Clinical Laboratory, Aichi Medical University Hospital, Nagakute, Aichi 480-1195, Japan; menomo@aichi-med-u.ac.jp (M.E.); tani@aichi-med-u.ac.jp (H.T.); 3 Department of Tumor Immunology, Aichi Medical University School of Medicine, Nagakute, Aichi 480-1195, Japan; suzukis@aichi-med-u.ac.jp; 4 Research Creation Support Center, Aichi Medical University, Nagakute, Aichi 480-1195, Japan; 5 Department of Hematology and Rheumatology, Faculty of Medicine, Kindai University, Osakasayama 589-8511, Japan; luis@med.kindai.ac.jp

**Keywords:** Gnetin-C, resveratrol dimer, cancer chemoprevention, serum biomarker, immunomodulation, uric acid

## Abstract

Gnetin-C is a naturally occurring stilbene derived from the seeds of *Gnetum gnemon L.*, an edible plant native to Southeast Asia that is called melinjo. Although the biological properties and safety of *G. gnemon* extract, which contains nearly 3% Gnetin-C, have been confirmed in various human studies, whether or not pure Gnetin-C is safe for humans is unclear at present. We conducted a randomized, double-blind, placebo-controlled trial. Healthy subjects were randomly divided into two groups. The interventional group (*n* = 6) was given Gnetin-C, and the control group (*n* = 6) was provided a placebo, for 14 days. Lipid profiles, biomarkers of oxidative stress and circulating blood cells were assessed before and after the intervention. All subjects completed the study, with no side effects reported across the study duration. Gnetin-C supplementation demonstrated a statistically significant increase in the absolute number of circulating natural killer (NK) cells expressing the activating receptors NKG2D and NKp46. NK cells derived from subjects who received Gnetin-C for two weeks showed higher cytotoxicity against K562 target cells than those before receiving Gnetin-C. In addition, Gnetin-C also resulted in a significant decrease in the absolute neutrophil count in the blood compared with the placebo. Furthermore, Gnetin-C significantly reduced the levels of uric acid, low-density lipoprotein cholesterol, high-density lipoprotein cholesterol, total adiponectin, and high-molecular-weight adiponectin. These data indicate that Gnetin-C has biological effects of enhancing the NK activity on circulating human immune cells. The immunomodulatory effects are consistent with a putative improvement in cancer immunosurveillance via the upregulation of the NKG2D receptor. The study was registered with UMIN-CTR, number 000030364, on 12 December 2017.

## 1. Introduction

Numerous preclinical and clinical studies have documented that resveratrol has several health promoting properties, including anti-inflammatory, antioxidant, and antitumor potential, however, the clinical utility of this naturally-occurring compound is hampered by its extensive liver metabolism and poor bioavailability [1]. As a result, several attempts to improve the bioavailability of this compound have been made. For example, the generation of synthetic resveratrol analogs [2], the use of bioactive resveratrol metabolites, or the utilization of naturally occurring or synthetic resveratrol oligomers with variable results [3].

Gnetin-C is a naturally-occurring stilbenoid, a dimer of resveratrol, that in recent years has attracted considerable scientific attention since this compound, like other resveratrol oligomers, has health-promoting properties that appear to be equal or even superior than those of resveratrol monomer [3,4]. Gnetin-C has been isolated from various tropical evergreen trees, shrubs, and lianas of the *Gnetaceae* family, such as *G. gnemon*, *G. leyboldii*, *G. schwackeanum*, and *G. africanum*, as well as from *Rheum lhasaense*, a plant of the *Polygonaceae* family [3,4]. A rich source of Gnetin-C is *Gnetum gnemon L.* (*G. gnemon*), melinjo, an edible plant native to Southeast Asia whose seeds and fruits are broadly used in Indonesian culinary [5]. It must be noted, however, that a number of preclinical and clinical studies assessing the biological properties of Gnetin-C have been conducted using *G. gnemon* extract, which in addition to Gnetin-C also contains various stilbenoids, including trans-resveratrol (resveratrol monomer), Gnetin-L, Gnemonoside-A, Gnemonoside-C, and Gnemonoside-D [5,6,7].

Data from preclinical studies indicate that *G. gnemon* possesses antioxidant and antimicrobial effects [5,8], tumor inhibitory properties [6,9], and anti-inflammatory immune modulatory activities [7,10,11]. In humans, high doses of *G. gnemon* powder (5000 mg daily) were well-tolerated [12], and a randomized controlled study showed a significant decrease in serum uric acid levels along with an increase in the plasma levels of high-density lipoprotein cholesterol (HDL-C) after *G. gnemon* consumption [13].

Purified Gnetin-C also possess anti-cancer properties. For example, in a mouse model of human acute myeloid leukemia (AML), Gnetin-C showed powerful anti-leukemia properties via inhibition of mTOR and MAPK kinases, two pathways that are aberrantly activated in AML cells [14]. In prostate cancer cells, Gnetin-C showed more potent tumor inhibitory effects than resveratrol or Pterostilbene. Mechanistically, Gnetin-C specifically and strongly downregulates metastasis-associated protein 1 (MTA1) and the ETS-proto-oncogene 2 (ETS2) expression in prostate cancer cells [15].

Although the safety of *G. gnemon* in humans has been consistently established, whether or not pure Gnetin-C is safe is unclear at present. In this study, we assessed the safety of Gnetin-C in healthy Japanese individuals and investigated the potential health-promoting effects of this compound by measuring the putative changes in circulating immune cells and in certain metabolic parameters in plasma and serum samples associated with Gnetin-C administration.

## 2. Materials and Methods

### 2.1. Study Design

A total of 12 healthy volunteers (male, *n* = 6; female, *n* = 6; median age, 29 years (range, 24–53 years); median body weight, 55 kg (range, 42–81 kg); median body length 162 cm (range, 154–175 cm); median body mass index (BMI), 18 kg/m^2^ (range, 14–27 kg/m^2^)) were enrolled in this phase 1 randomized trial (UMIN-CTR #UMIN000030364), which was approved by the Aichi Medical University Hospital Institutional Review Board (#2017-H227) and conducted in accordance with the Declaration of Helsinki. All subjects provided their written informed consent to participate in this study. The inclusion criteria were as follows: the absence of subjective symptoms, a normal liver and renal function, drinking alcohol fewer than four times per week and a willingness to abstain from the ingestion of resveratrol-containing foods and drink. The exclusion criteria were as follows: the long-term use of medication, chronic disease, a history of invasive cancer within the past five years, a history of non-invasive cancer within the past year, and a history of smoking within the past year.

To control potential spontaneous changes in immune cells that were not associated with the administration of Gnetin-C, the study included a group of six healthy subjects who were given the same instructions as the Gnetin-C group with regard to refraining from ingesting wine or Gnetin-C -rich foods and who were willing to provide blood samples at baseline and days 7, 14, and 28 after study entry. The Gnetin-C powder was directly extracted and purified from *G. gnemon* and contained 26.3% Gnetin-C, 51.1% dextrin, and 22.6% sucrose fatty acid ester as an emulsifier. Capsules containing 210 mg each of the Gnetin-C powder and those of the placebo powder were donated by HOSODA SHC Co., Ltd. (Fukui, Japan). The enrolled subjects were randomized at a 1:1 ratio to ingest 3 capsules daily of 150 mg Gnetin-C net (Gnetin-C group; *n* = 6) or 3 capsules daily of the placebo (placebo-control group; *n* = 6).

The subjects were instructed to ingest Gnetin-C or the placebo after lunch daily for 14 days, and were evaluated on a weekly basis to investigate their compliance and the incidence of adverse events according to the National Cancer Institute Common Terminology Criteria for Adverse Events (CTCAE) version 4.0 [16]. The primary objective of this trial was to evaluate the safety of Gnetin-C in Japanese individuals. The secondary objective was to assess the potential effects of Gnetin-C on circulating lymphocytes and on various metabolic serological parameters.

### 2.2. Sample Preparation and Pharmacokinetic Evaluations

Blood samples (~20 mL) were collected in blood collection tubes with or without EDTA2Na at baseline and days 7, 14, and 28 after study entry. From each sample, 7 mL of blood with EDTA2Na was centrifuged (20 °C, 10 min, 3000 g) to isolate plasma, and 8 mL of blood collected in blood collection tubes without EDTA2Na was centrifuged under refrigeration (8 min, 3000 g) to isolate serum. To prevent potential light-based damage to Gnetin-C, its metabolites, and to other blood metabolic markers, the plasma and serum were transferred to 1.5 mL LightSafe centrifuge tubes and stored at −80 °C until use.

Peripheral blood mononuclear cells (PBMCs) were isolated from the remaining 14 mL of the blood using gradient centrifugation. A fraction of the isolated PBMCs from each sample was immediately analyzed by flow cytometry to assess the lymphocyte populations and the expression of relevant surface markers, the other fraction was cryopreserved for further use. A high-performance liquid chromatography (HPLC) analysis for the quantification of Gnetin-C was performed, as described in a previous study [12].

### 2.3. Whole Blood Cell Counts

Whole blood cell counts and individual leukocyte fractions were analyzed using an XN-9000 (Sysmex Corporation, Kobe, Hyogo, Japan) by flow cytometry. Absolute neutrophil counts were calculated by multiplying the leukocyte count by the neutrophil fraction.

### 2.4. Flow Cytometry

Freshly isolated PBMCs were stained with antibodies specific to the cell surface markers of T cells, B cells, and NK lymphocyte, including anti-CD3, CD19, CD20, CD4, CD8, CD16, CD56, NKp46, NKG2D, NKp30, TLR4, DNAM-1, NKG2A, 4–1BB, OX40, ICOS, PD-1, CTLA4, GITR, LAG3, TIGIT, and TIM3 (BioLegend, San Diego, CA, USA). The stained cells were analyzed using a FACSCant II (BD Biosciences, San Jose, CA, USA), and the data were analyzed using the FlowJo software package (ver.10; Tree Star, Ashland, OR, USA).

### 2.5. Cytotoxicity Assay

K562 cells were used as target cells for NK cell-mediated cytotoxicity, as assessed using an Annexin V affinity assay. K562 cells were maintained in culture medium containing ALys505N-O supplemented with 10% FCS. K562 cells in the log phase of growth were harvested by centrifugation at 800 rpm for 10 min at room temperature (RT). The supernatant was discarded, and the cells were resuspended at a concentration of 2 × 10^4^ cells/mL in culture medium. Cryopreserved PBMCs were thawed at 37 °C for 5 min and harvested by centrifugation at 1200 rpm for 3 min at room temperature. The supernatant was discarded, and the cells were resuspended in 80 µL auto macs rinsing solution. Anti-CD3 microbeads (20 µL), anti-CD14 microbeads (20 µL), and anti-CD19 microbeads (20 µL) were added and incubated for 15 min at 4 °C. Cells were diluted in 2 mL auto macs rinsing solution, pelleted, resuspended in 500 µL auto macs rinsing solution, and magnetically separated using an Auto MACS (MACS Militenyi Biotec, Tokyo, Japan). Flow-through and 2 mL of solution containing NK cells as negative fraction, were harvested and centrifugated at 1200 rpm for 3 min at room temperature. The supernatant was discarded, and the cells were resuspended at a concentration of 5 × 10^4^ cells/mL in culture medium.

The effector cells (NK cells) were co-incubated with target cells (K562) at a 2.5:1, 1.25:1, and 0.6:1 effector:target ratio (E:T) for 18 h. NK cell cytotoxicity against K562 was determined by flow cytometry. These cells were stained with antibodies specific to the cell surface markers of NK cells and K562, including anti-CD3, CD56, and Glycophorin A (BioLegend, San Diego, CA, USA). The stained cells were analyzed using a FACSCanto II instrument (BD Biosciences), and the data were analyzed using the FlowJo software package.

### 2.6. Serum Metabolic Profile

The serum concentrations of metabolic markers and biochemical substances, including low-density lipoprotein cholesterol (LDL-C), high-density lipoprotein cholesterol (HDL-C), uric acid, total protein, albumin, total bilirubin, serum creatinine, urea nitrogen, aspartate transaminase, alanine aminotransferase, lactate dehydrogenase, γ-glutamyltransferase, triglyceride, and C-reactive protein, were analyzed using a LABOSPECT008 (Hitachi-Technologies Corporation, Tokyo, Japan) with a colorimetric assay. Adiponectin was measured by the latex immunity nephelometry measurement method (at SRL Inc, Tokyo, Japan).

### 2.7. The Measurement of Urinary 8-Oxo-2’-Deoxyguanosine (8-OHdG)

Urine samples were collected from the participants in sterile tubes at baseline and at days 7, 14, and 28 of Gnetin-C administration. The samples were always kept refrigerated, and the assessment of the urinary levels of Urinary 8-Oxo-2’-Deoxyguanosine (8-OHdG), adjusted by the creatinine concentration correction method, was performed at the Japan Institute for the Control of Aging (JaICA; Nikken Sail Co. Ltd, Fukuroi, Japan).

### 2.8. The Measurement of Pentosidine (Advanced Glycation Endroducts, AGEs)

Pentosidine (as an Advanced Glycation Endroducts (AGE) biomarker) was measured by an enzyme-linked immunosorbent assay (ELISA) at JaICA Nikken Sail Co. Ltd.

### 2.9. Statistical Analyses

Data are reported as the mean ± standard deviation. Some comparisons in this study were based on the fold change, which was calculated as the ratio of difference between the final value (value at a given time) and the initial value (baseline level) over the initial value. Thus, if the baseline value was A and final value was B, the fold change was (B-A)/A. When comparisons were made between two different groups, the statistical significance was determined using Student’s *t*-test. The statistical significance of multiple comparisons was determined using a one-way analysis of variance. *p* values of ≤0.05 were considered to indicate statistical significance.

## 3. Results

### 3.1. The Safety and Pharmacokinetic Evaluations

**Figure 1:**
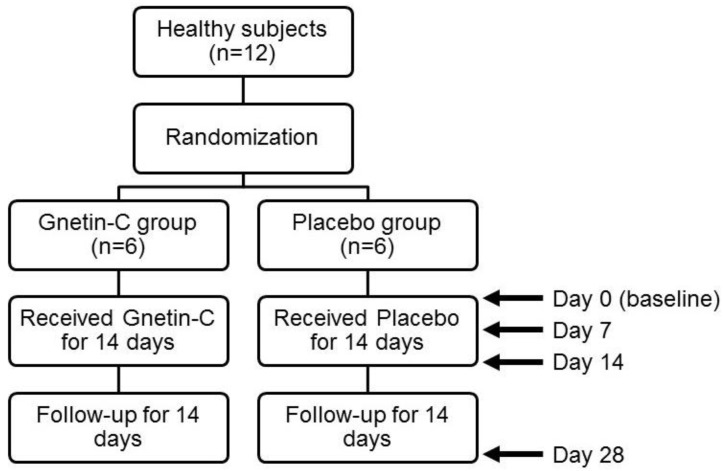
Flow diagram of the study.

Twelve healthy subjects (Table 1) were enrolled in this study, stratified by gender, randomized, and assigned to receive 3 capsules of Gnetin-C daily (150 mg/day) or a placebo for 14 consecutive days (the test period), followed by an observational period for the next 14 days (Figure 1). No adverse events were reported during the test or observational period.

**Figure 2:**
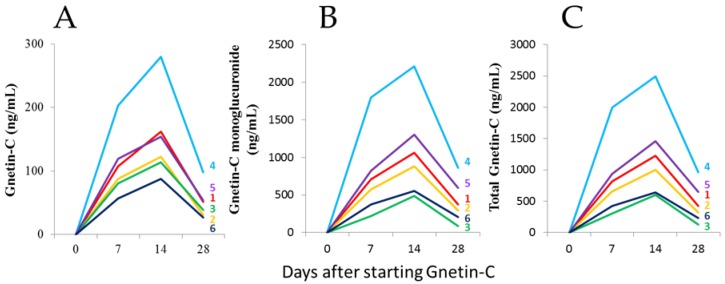
(**A**) Plasma concentrations of Gnetin-C; (**B**) the Gnetin-C metabolite, Gnetin-C monoglucuronide; (**C**) total Gnetin-C before and after Gnetin-C supplementation. In (**A**–**C**), subject 1 is shown in red, subject 2 in orange, subject 3 in green, subject 4 in light blue, subject 5 in purple, and subject 6 in blue.

The plasma Gnetin-C concentrations measured by a high-performance liquid chromatography (HPLC) analysis were (Figure 2A) 109 ± 51 ng/mL (range, 57–203 ng/mL) at day 7, 153 ± 68 ng/mL (range, 87–279 ng/mL) at day 14, and 50 ± 26 ng/mL (range, 27–98 ng/mL) at day 28. (Figure 2B) The concentrations of the Gnetin-C metabolite Gnetin-C monoglucuronide, were 748 ± 558 ng/mL (range, 221–1797 ng/mL) at day 7, 1083 ± 632 ng/mL (range, 487–2210 ng/mL) at day 14, and 402 ± 283 ng/mL (range, 86–862 ng/mL at day 28. (Figure 2C) The total Gnetin-C concentrations were 857 ± 608 ng/mL (range, 302–1999 ng/mL) at day 7, 1236 ± 698 ng/mL (range, 601–2490 ng/mL) at day 14, and 452 ± 306 ng/mL (range, 124–960 ng/mL at day 28. As expected, Gnetin-C was not detected in any of the plasma samples collected at baseline (day 0) or in the samples derived from the placebo group.

### 3.2. The Effects of Gnetin-C on Whole Blood Cell Counts

**Figure 3:**
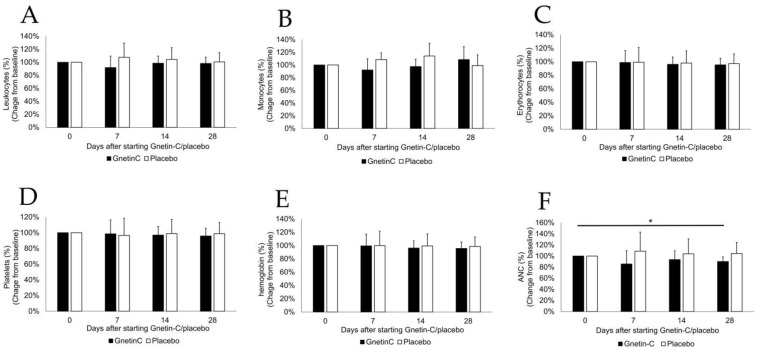
The effects of Gnetin-C on whole blood cell counts. No significant changes were noted in the numbers of (**A**) leukocytes; (**B**) monocytes; (**C**) erythrocytes; or (**D**) platelets; or (**E**) in the hemoglobin concentration, in association with the Gnetin-C intake. However, (**F**) Gnetin-C administration resulted in a significant decrease in the absolute neutrophil count (ANC) in the blood.

To assess the effects of the repeated administration of Gnetin-C in healthy individuals, we first performed whole blood cells counts at baseline and after the administration of Gnetin-C or placebo using a blood cell counter. As shown in Figure 3A–E, we observed no significant changes in the numbers of leukocytes, monocytes, erythrocytes, or platelets or in the hemoglobin concentration associated with the Gnetin-C intake. However, Gnetin-C administration resulted in a significant decrease in the absolute neutrophil count (ANC) in the blood (86% ± 24% at day 7, *p* = 0.24; 94% ± 16% at day 14, *p* = 0.41; 90% ± 8% at day 28, *p* = 0.045; Figure 3F).

### 3.3. The Effects of Gnetin-C on Circulating Peripheral Blood Mononuclear Cells (PBMCs)

**Figure 4:**
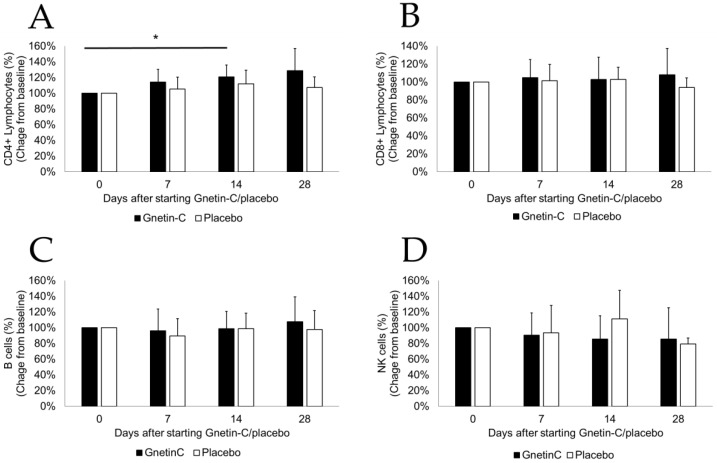
The effects of Gnetin-C on circulating peripheral blood mononuclear cells (PBMCs) were examined by flow cytometry. The administration of Gnetin-C resulted in a significant increase in the (**A**) CD4 + T cell population; (**B**) Regarding other circulating lymphocytes, including CD8 + T cells; (**C**) CD20 + B cells, and (**D**) NK cells, no marked changes were noted. * *p* < 0.05.

Flow cytometry of PBMCs from blood samples collected at baseline (reference) and at days 7, 14, and 28 revealed a statistically significant increase in the CD4 + T cells (114% ± 16% at day 7, *p* = 0.102; 121% ± 15% at day 14, *p* = 0.026; 129% ± 28% at day 28, *p* = 0.072; Figure 4A). Of note, the administration of Gnetin-C did not result in changes in other circulating lymphocytes, including CD8 + T cells, CD20 + B cells, and NK cells (Figure 4B–D).

### 3.4. Effects of Gnetin-C on PBMC Subsets

**Figure 5:**
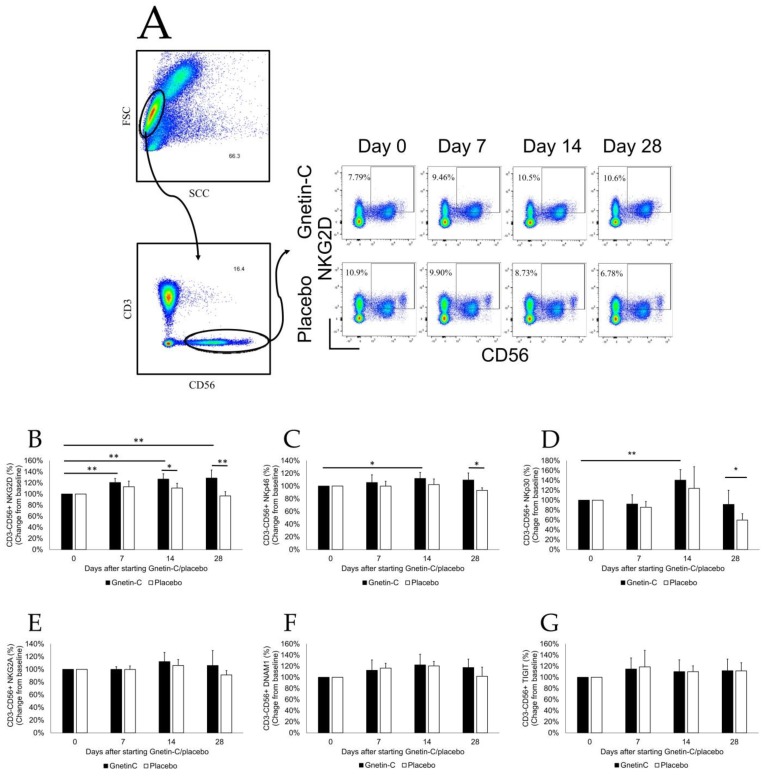
The effects of Gnetin-C on circulating peripheral blood mononuclear cells (PBMCs). (**A**) A representative flow cytometry scattergram indicating the increased NKG2D expression in NK cells obtained from healthy subjects after Gnetin-C supplementation; PBMCs were assessed by flow cytometry for the expression of cell surface markers at baseline and after the intervention. (**B**) The administration of Gnetin-C resulted in a significant increase in the activator receptors NKG2D; (**C**) NKp46, and (**D**) NKp30 at the cell surface of NK cells. Other cell surface markers of NK cells; including (**E**) NKG2A; (**F**) DNAM1, and (**G**) TIGIT, did not change markedly during the study. * *p* < 0.05; ** *p* < 0.01.

Flow cytometry of PBMCs from blood samples collected at baseline (reference) and at days 7, 14, and 28 revealed a statistically significant increase in the absolute number of circulating NK cells expressing the activating receptors NKG2D (121% ± 7% at day 7, *p* = 0.0015; 127% ± 10% at day 14, *p* = 0.0015; 129% ± 15% at day 28, *p* = 0.0071; Figure 5A,B), NKp46 (106% ± 12% at day 7, *p* = 0.35; 112% ± 10% at day 14, *p* = 0.040; 109% ± 11% at day 28, *p* = 0.11; Figure 5C) and NKp30 (92% ± 19% at day 7, *p* = 0.39; 140% ± 21% at day 14, *p* = 0.0082; 91% ± 29% at day 28, *p* = 0.53; Figure 5D). Of note, the administration of Gnetin-C did not result in changes in the number of circulating NK cells expressing other receptors, including NKG2A (Figure 5E), DNAM1 (Figure 5F), TIGIT (Figure 5G), TLR4, 4–1BB, OX40, ICOS, PD-1, CTLA4, GITR, LAG3, and TIM3.

### 3.5. Effect of Gnetin-C on NK Cell-Mediated Cytotoxicity

**Figure 6:**
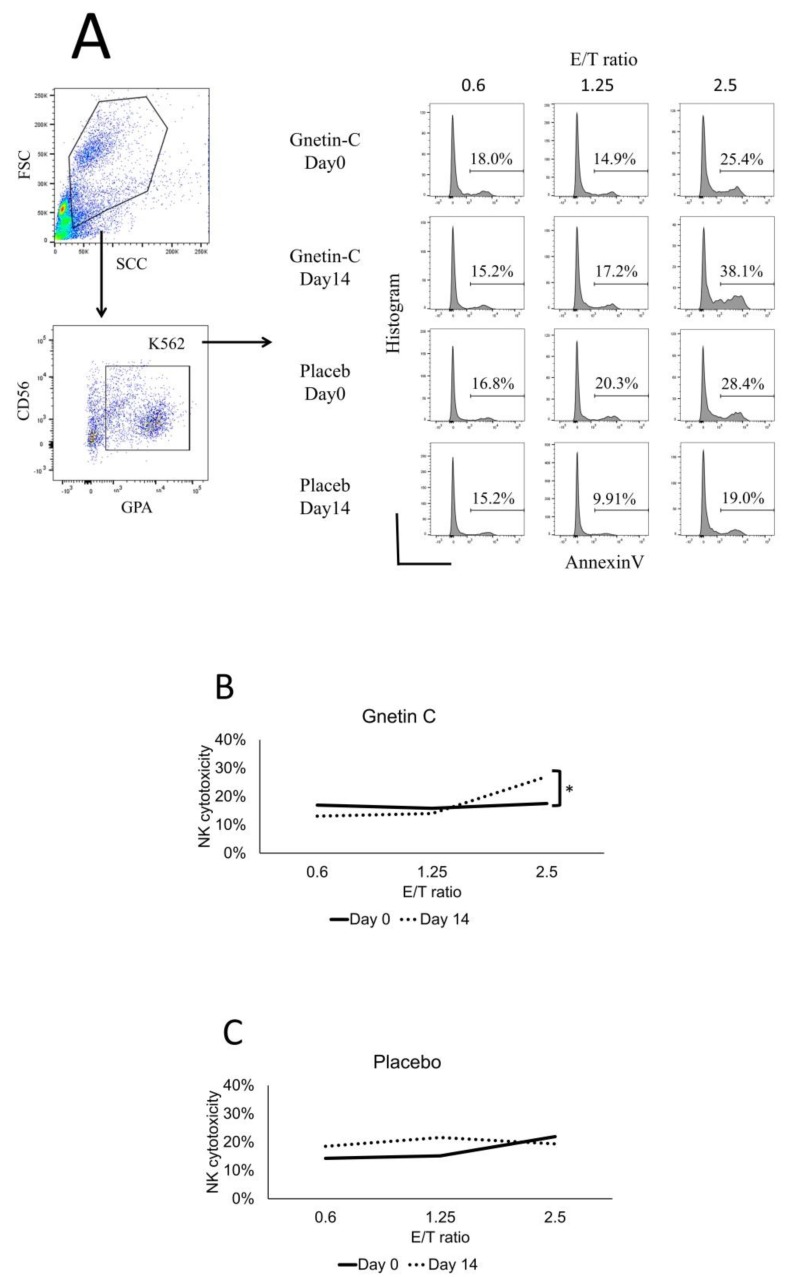
(**A**) The cytotoxicity of NK cells against K562 cells in Gnetin-C; A representative flow cytometry scattergram indicating the increase in the proportion of apoptotic K562 cells (as indicated by Annexin V expression) when exposed to NK cells obtained from healthy subjects after Gnetin-C supplementation and placebo. Summarized cytotoxicity data of NK cells derived from individuals in the (**B**) Gnetin-C group and (**C**) placebo group, before and after the intervention, as determined by an Annexin V affinity assay. * *p* < 0.05.

We conducted functional studies to assess whether or not the administration of Gnetin-C affected the cytotoxic activity of NK cells. We performed an in vitro killing assay, in which cryopreserved PBMCs collected before and after Gnetin-C or placebo intake were co-cultured with K562 cells, a leukemia cell line that lacks human leukocyte antigen (HLA) class one (HLA-I) on its surface and can thus be utilized as a standard target of NK cells cytotoxicity. As shown in Figure 6, NK cells derived from subjects who received Gnetin-C for 14 days consistently showed higher cytotoxicity against K562 target cells than cells obtained before the subjects had received Gnetin-C, while these effects were not seen in NK cells derived from subjects who had received the placebo (Figure 6B,C).

### 3.6. The Effects of Gnetin-C on Biochemical Tests and Lipid Profiles

**Figure 7:**
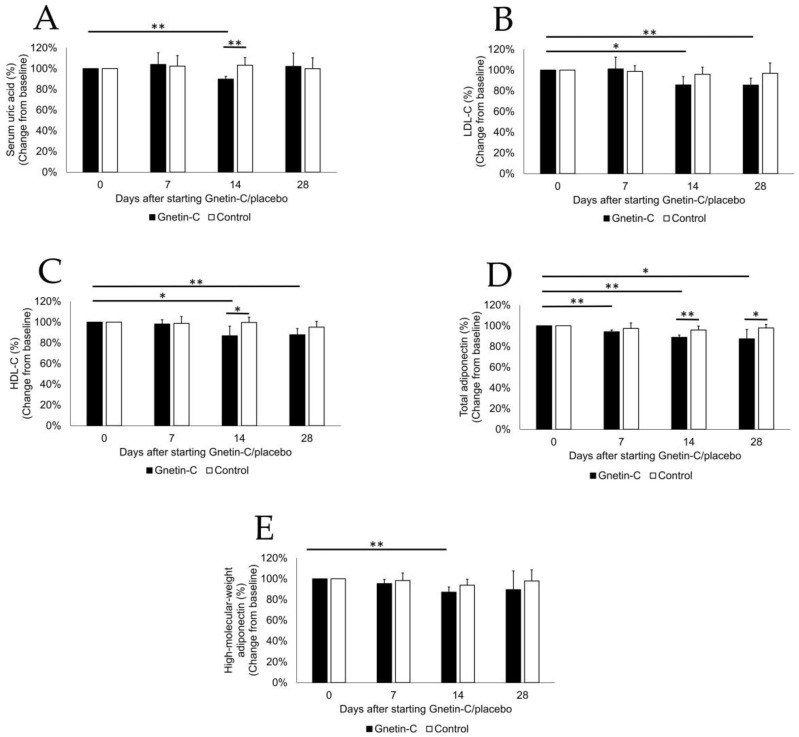
The effects of Gnetin-C on biochemical test results and lipid profiles. (**A**) Serum levels of uric acid; (**B**) low-density lipoprotein cholesterol (LDL-C); (**C**) high-density lipoprotein cholesterol (HDL-C); (**D**) total adiponectin, and (**E**) high-molecular-weight (HMW) adiponectin before and after the intervention in Gnetin-C and placebo groups. * *p* < 0.05; ** *p* < 0.01.

The administration of Gnetin-C was associated with significant decreases in the serum levels of uric acid at day 14 after starting Gnetin-C supplementation (104% ± 11% at day 7, *p* = 0.46; 90% ± 10% at day 14, *p* = 0.00036; 102% ± 13% at day 28, *p* = 0.72; Figure 7A), and this was also seen in comparison with the levels observed in the placebo group (103% ± 7% at day 14, *p* = 0.0032). In contrast, we observed a decrease in the serum levels of LDL-C (101% ± 11% at day 7, *p* = 0.84; 86% ± 8% at day 14, *p* = 0.011; 86% ± 7% at day 28, *p* = 0.0047; Figure 7B) as well as in HDL-C (98% ± 4% at day 7, *p* = 0.38; 87% ± 9% at day 14, *p* = 0.025; 88% ± 6% at day 28, *p* = 0.0062; Figure 7C), along with a consistent decrease in the serum levels of adiponectin both total (94% ± 2% at day 7, *p* = 0.00054; 89% ± 2% at day 14, *p* = 0.000087; 87% ± 9% at day 28, *p* = 0.026; Figure 7D) and high-molecular-weight (95% ± 4% at day 7, *p* = 0.051; 87% ± 5% at day 14, *p* = 0.0021; 90% ± 18% at day 28, *p* = 0.25; Figure 7E) forms compared with the baseline levels as well as the levels observed in the placebo group. These effects were still noticeable two weeks after taking the last dose of Gnetin-C in most cases. Other biochemical tests, including assessments of total protein, albumin, total bilirubin, serum creatinine, urea nitrogen, aspartate transaminase, alanine aminotransferase, lactate dehydrogenase, γ-glutamyltransferase, triglyceride, and C-reactive protein, showed no significant difference in the serum levels between the two groups (Table 2).

### 3.7. The Effects of Gnetin-C on Urinary 8-OHdG and Pentosidine

**Figure 8:**
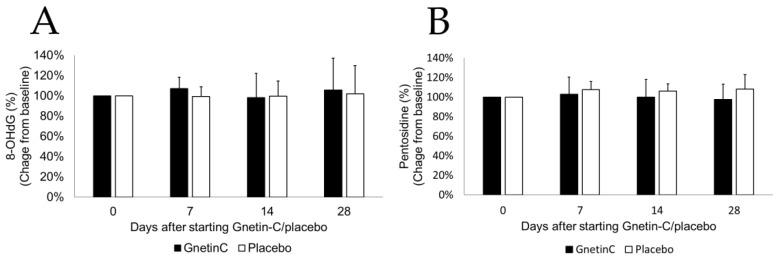
The effects of Gnetin-C on 8-Oxo-2′-deoxyguanosine and pentosidine. (**A**) 8-Oxo-2′-deoxyguanosine and (**B**) pentosidine before and after the intervention in Gnetin-C and placebo groups.

We next assessed the urinary levels of 8-OHdG, a systemic marker of oxidative stress that is downregulated in subjects receiving resveratrol [17]. In this study, however, the administration of Gnetin-C did not result in any marked changes in urinary 8-OHdG or pentosidine (Figure 8A,B), thus confirming that resveratrol and Gnetin-C have different biological effects in humans.

## 4. Discussion

In this study we verified that pure Gnetin-C given at 150 mg/day for two weeks is safe in humans. Our data also revealed that the administration of this compound for two weeks resulted in measurable effects in circulating immune cells. In addition, Gnetin-C supplementation was associated with detectable changes in certain metabolic markers, including a decrease in the levels of uric acid, as well as significant reductions in the levels of both LCL-C and HDL-C and plasma levels of adiponectin.

As mentioned above, Gnetin-C is a dimer of resveratrol, and given its variable effects on immune cells, resveratrol can be considered an immunomodulatory molecule [18], which has been documented in human studies showing that the administration of resveratrol induces measurable changes in circulating immune cells. For example, resveratrol given for up to four weeks in healthy individuals induced a modest but significant increase in the number of regulatory T cells (Treg) [17], which can in part explain the anti-inflammatory properties of this compound, since Treg cells play a pivotal role in preventing auto-immunity [19]. *G. gnemon* (which contains Gnetin-C and other resveratrol oligomers) may also have immunomodulatory effects, as suggested by preliminary studies, although the precise compound within the *G. gnemon* mixture capable of exerting those effects has remained unclear [7,11]. The findings that Gnetin-C modulates immune cells in vivo may therefore provide further evidence that dietary phytochemicals directly or indirectly modulate the immune system and are consistent with the proposed cancer chemopreventive properties of some of those compounds [20,21,22].

Of particular interest is the finding that NK cells, especially NK subsets expressing the NKG2D receptor on their surface, are significantly upregulated after Gnetin-C administration. NK cells are an essential component of the innate immune system that play a major role in the elimination of virus-infected and malignant cells. They express multiple activator receptors on their cell surface that are required for the recognition of specific ligands on the potential target cells. Among those receptors, NKG2D is critical for cancer immunosurveillance [23], since immune cells expressing this receptor recognize and eliminate target cells expressing specific ligands (MICA/B and ULBP1/2/3/4/5/6 ligands) that are upregulated in cells exposed to stress stimuli, such as after viral infection, heat shock, and malignant transformation [24,25]. The fact that Gnetin-C upregulates NKG2D-expressing NK cells is consistent with the notion that this compound may enhance the cancer-preventive properties of immune cells.

Previous studies [26,27,28,29] have shown that a monomer of resveratrol enhances NK cytotoxicity in vitro through the upregulation of the activating receptor NKG2D. The present study showed that Gnetin-C, a dimer of resveratrol, enhances the NKG2D expression and NK cytotoxicity as well. Furthermore, this is the first study to show that such effects actually develop in circulating NK cells of humans after taking resveratrol, regardless of whether it is a monomer or a dimer of resveratrol.

Our previous study [7] showed that *G. gnemon* has an immunomodulatory effect on NK activity in healthy individuals, with which the current results were consistent. The Gnetin-C purity in *G. gnemon* used in the previous study [7] was only 2.5%, and it was mixed with various stilbenoids, such as trans-resveratrol (resveratrol monomer), Gnetin-L, Gnemonoside-A, Gnemonoside-C, and Gnemonoside-D, thus making it unclear whether the immunomodulatory effect should be attributed to Gnetin-C alone. However, this study did reveal for the first time that Gnetin-C truly has an immunomodulatory effect in vivo because pure Gnetin-C was used.

Our previous study [14] showed that Gnetin-C induces apoptosis in acute myeloid leukemia (AML) through simultaneously inhibiting the mTOR and MAPK pathways, which are essential for the survival of leukemia cells. Gnetin-C actually attenuated the formation of leukemia, depleted leukemia cells, and improved the survival in experiments using human leukemia-model mice. The present study revealed that Gnetin-C supplementation safely enhances the NKG2D expression and NK cytotoxicity in healthy individuals, although whether or not Gnetin-C affects the mTOR and MAPK pathways and their interaction with the NK cell function in healthy individuals remained unclear, as we lacked available samples for an investigation. Leukemia cells can be eliminated by the upregulation of the NKG2D/NKG2D-ligand system [30]. Therefore, Gnetin-C has attractive therapeutic potential against AML and may induce both direct and indirect anti-leukemia effects, leading to the efficient eradication of AML cells. We are now planning clinical trials using Gnetin-C for AML patients who are not eligible for standard chemotherapy and those who are in complete remission after chemotherapy but not eligible for allogeneic hematopoietic stem cell transplantation.

The uric acid levels being reduced after Gnetin-C intake in the present study is a somewhat expected finding, since Konno et al. showed in a previous study conducted in healthy Japanese individuals that *G. gnemon* supplementation resulted in a substantial decrease in the levels of uric acid by inhibiting the reabsorption of this metabolic waste in the renal tubular epithelia [13]. Therefore, the present study may indicate that Gnetin-C is very likely the component within the *G. gnemon* that is responsible for lowering the uric acid levels in humans. Similarly, the monomeric molecule resveratrol was also reported to lower uric acid levels in preclinical studies [31,32], although, to our knowledge, these effects have not been documented in humans.

Of note, in their study, Konno et al. reported a significant increase in the levels of HDL-C associated with *G. gnemon* supplementation, without noticeable changes in the levels of LDL-C. These observations were in apparent disagreement with the findings of the present study, where significantly lower levels of both HDL-C and LDL-C were observed at two weeks after Gnetin-C intake. These discrepancies may be attributed of the fact that, contrary to *G. gnemon*, pure Gnetin-C is capable of decreasing levels of all lipoproteins, regardless of their molecular weight. The difference in the study design may also be responsible for these contradictory results. For example, while subjects enrolled in the study by Konno et al. took *G. gnemon* for eight weeks, in the present study, individuals received Gnetin-C for only two weeks. In addition, although the metabolic changes (decrease in uric acid levels and increase in HDL-C levels) reported in Konno′s study were still detectable at week four of the intervention, the most significant changes were noted at week eight of study entry. One may speculate that the effects of Gnetin-C on lipoproteins and adiponectin may be most noticeable during acute supplementation and might disappear with extended exposure to these agents. Alternatively, pure Gnetin-C may act as a dietary phytochemical capable of interfering with the intestinal absorption of cholesterol and thereby may indirectly contribute to the reduction in the serum levels of cholesterol. Indeed, thus far, numerous dietary-derived phytochemicals have been described and their potential utility for preventing cardiovascular disorders associated with metabolic syndrome is an area of active research [33].

Because of its poor water solubility, cholesterol is packaged within lipoproteins and is transported through the blood via emulsification. LDL-Cs are the major blood cholesterol carriers and promote the deposit of excess cholesterol in peripheral tissues, especially in the blood vessels, which eventually promotes the development of atheromatous plaques. Conversely, HDL-Cs transport cholesterol back to the liver, even cholesterol molecules that have been deposited in atherosclerotic lesions of blood vessels, thus counteracting the deleterious effect of LDL-C in the development of cardiovascular disorders associated with lipid imbalance and metabolic syndrome. In fact, increased plasma levels of HDL-C correlate with better health outcomes, whereas low numbers of HDL particles are associated with the poorest outcomes, particularly with regard to the progression of atherosclerosis [34]. Based on these concepts, one may speculate that Gnetin-C consumption may promote the development of atherosclerosis, thus increasing the cardiovascular risk. However, factors associated with increased cardiovascular risk, in the setting of lipid disorders, are known to require long-term exposure [35]. Given that we assessed the effects of Gnetin-C supplementation for only two weeks in the current study, the short exposure time might have impeded any categorical conclusions about the long-term impact of these findings on the metabolic profile. Therefore, further studies enrolling larger populations, followed for a longer period of time, are needed to determine the effects of Gnetin-C on metabolic markers in humans.

Another relevant finding of this study was that levels of total adiponectin and high-molecular-weight adiponectin decreased significantly in individuals taking Gnetin-C. Human adiponectin is a hormone protein encoded by the *ADIPOQ* gene that is produced almost exclusively in adipose tissue and is involved in the regulation of glucose levels and fatty acid metabolism. Functionally, adiponectin plays a role in the suppression of body disturbances coupled with metabolic syndrome, such as obesity, atherosclerosis, non-alcoholic fatty liver disease, and diabetes, and thus it exerts a wide range of beneficial effects on the cardiovascular system [36]. In this regard, reduced adiponectin levels have been reported to pose an increased risk of cardiovascular complications in cases of obesity, insulin resistance, and diabetes. However, adiponectin levels are markedly increased in individuals with advanced cardiovascular disorders such as heart failure [37]. Paradoxically, circulating adiponectin, both total and high-molecular-weight forms, are positively associated with the mortality rate, not only in the presence of cardiovascular disease but also across all clinical settings [38]. Therefore, since the clinical significance of adiponectin as a biomarker in cardiovascular diseases remains controversial, the implications of decreased levels of adiponectin associated with Gnetin-C intake, as we observed in the present study, are currently unknown.

In previous studies, the anti-leukemia activities of Gnetin-C were observed at concentrations as low as 2.5 μM (1136 ng/mL) [14] and peaked at 20 μM. Interestingly, in the present study, total Gnetin-C in plasma samples collected at day 14 was detectable at concentrations ranging from 601 to 2490 ng/mL, suggesting that biologically active concentrations with potential anticancer effects could be achieved in vivo. This is particularly important considering that Gnetin-C was well-tolerated, and in a putative therapeutic setting, this agent could be given at doses higher than the doses used in this study. Furthermore, taking into account that Gnetin-C was given for only 14 days, the fact that considerable amounts of pure Gnetin-C (range, 27–98 ng/mL) and its monoglucuronide metabolite (range, 86–862 ng/mL) were still detectable in blood samples collected at day 28 of study entry (14 days after the last dose) indicates that Gnetin-C persists for some days in the body, likely deposited in tissues from where it recirculates into the blood stream. These findings are consistent with those of previous studies showing that Gnetin-C has a higher bioavailability than resveratrol [12,39].

Limitations associated with the present study include the small size and the relatively short exposure of the subjects to Gnetin-C. The use of a placebo group allowed us to verify that changes observed in circulating immune cells were in association with the intervention and not the result of inter-individual or inter assay variability. Further studies are warranted to investigate the metabolic effects of pure Gnetin-C in humans.

## Figures and Tables

**Table 1:** Demographic baseline characteristics of the enrolled subjects.

Group	Gnetin-C	Placebo	*p*
*n*	6	6	
M/F	3/3	3/3	
Median age, years (range)	34 (26–53)	27.5 (24–42)	0.26
Median height, m (range)	1.62 (1.57–1.75)	1.65 (1.54–1.73)	0.91
Median weight, kg (range)	55 (43–81)	59 (42–72)	0.90
Median body mass index, kg/m^2^ (range)	18.1 (14.3–26.9)	19.6 (14.0–23.9)	0.90

**Table 2:** The Effects of Gnetin-C on Biochemical Tests for 28 Days in Healthy Volunteers.

	Day 7		Day 14		Day 28		
	Gnetin C (n = 6)	Placebo (n = 6)	Gnetin C (n = 6)	Placebo (n = 6)	Gnetin C (n = 6)	Placebo (n = 6)	*P*
	Means ± SD	Means ± SD	Means ± SD	Means ± SD	Means ± SD	Means ± SD	
Total proteins (g/dL)	99% ± 4%	100% ± 2%	96% ± 4%	99% ± 2%	94% ± 4%	100% ± 2%	0.14
Albumin (g/dL)	99% ± 4%	100% ± 2%	97% ± 5%	99% ± 3%	95% ± 5%	99% ± 2%	0.36
Total bilirubin (mg/dL)	113% ± 27%	91% ± 20%	111% ± 36%	93% ± 31%	105% ± 30%	104% ± 25%	0.43
Serum creatinine (mg/dL)	102% ± 4%	96% ± 2%	100% ± 8%	98% ± 4%	102% ± 5%	103% ± 4%	0.63
Urea nitrogen (mg/dL)	102% ± 6%	102% ± 18%	96% ± 11%	105% ± 12%	92% ± 19%	102% ± 8%	0.24
Aspartate transaminase (U/L)	103% ± 18%	96% ± 12%	99% ± 13%	97% ± 9%	97% ± 12%	95% ± 5%	0.81
Alanine aminotransferase (U/L)	111% ± 23%	107% ± 17%	93% ± 22%	101% ± 16%	95% ± 18%	108% ± 15%	0.54
Lactate dehydrogenase (U/L)	96% ± 6%	97% ± 6%	95% ± 6%	93% ± 4%	95% ± 9%	96% ± 3%	0.76
γ-glutamyltransferase (U/L)	94% ± 13%	94% ± 7%	84% ± 15%	91% ± 11%	85% ± 20%	91% ± 6%	0.37
Triglyceride (mg/dL)	90% ± 13%	92% ± 19%	107% ± 37%	85% ± 15%	108% ± 30%	106% ± 27%	0.25
C-reactive protein (mg/dL)	87% ± 21%	175% ± 214%	242% ± 318%	133% ± 57%	132% ± 113%	129% ± 100%	0.47

Values are change from baseline. *P* for interaction of Day 14.

## References

[1] Espinoza J.L., Kurokawa Y., Takami A. (2019). Rationale for assessing the therapeutic potential of resveratrol in hematological malignancies. Blood Rev..

[2] Snyder S.A., Gollner A., Chiriac M.I. (2011). Regioselective reactions for programmable resveratrol oligomer synthesis. Nature.

[3] Espinoza J.L., Inaoka P.T. (2017). Gnetin-C and other resveratrol oligomers with cancer chemopreventive potential. Ann. N. Y. Acad. Sci..

[4] Keylor M.H., Matsuura B.S., Stephenson C.R. (2015). Chemistry and Biology of Resveratrol-Derived Natural Products. Chem. Rev..

[5] Kato E., Tokunaga Y., Sakan F. (2009). Stilbenoids isolated from the seeds of Melinjo (*Gnetum gnemon* L.) and their biological activity. J. Agric. Food Chem..

[6] Narayanan N.K., Kunimasa K., Yamori Y., Mori M., Mori H., Nakamura K., Miller G., Manne U., Tiwari A.K., Narayanan B. (2015). Antitumor activity of melinjo (*Gnetum gnemon* L.) seed extract in human and murine tumor models in vitro and in a colon-26 tumor-bearing mouse model in vivo. Cancer Med..

[7] Espinoza J.L., An D.T., Trung L.Q., Yamada K., Nakao S., Takami A. (2015). Stilbene derivatives from melinjo extract have antioxidant and immune modulatory effects in healthy individuals. Integr. Mol. Med..

[8] Watanabe K., Shibuya S., Ozawa Y., Izuo N., Shimizu T. (2015). Resveratrol Derivative-Rich Melinjo Seed Extract Attenuates Skin Atrophy in Sod1-Deficient Mice. Oxid. Med. Cell. Longev..

[9] Kunimasa K., Ohta T., Tani H., Kato E., Eguchi R., Kaji K., Ikeda K., Mori H., Mori M., Tatefuji T. (2011). Resveratrol derivative-rich melinjo (*Gnetum gnemon* L.) seed extract suppresses multiple angiogenesis-related endothelial cell functions and tumor angiogenesis. Mol. Nutr. Food Res..

[10] Ikeda E., Ikeda Y., Wang Y., Fine N., Sheikh Z., Viniegra A., Barzilay O., Ganss B., Tenenbaum H.C., Glogauer M. (2018). Resveratrol derivative-rich melinjo seed extract induces healing in a murine model of established periodontitis. J. Periodontol..

[11] Kato H., Samizo M., Kawabata R., Takano F., Ohta T. (2011). Stilbenoids from the melinjo (*Gnetum gnemon* L.) fruit modulate cytokine production in murine Peyer’s patch cells ex vivo. Planta Med..

[12] Tani H., Hikami S., Iizuna S., Yoshimatsu M., Asama T., Ota H., Kimura Y., Tatefuji T., Hashimoto K., Higaki K. (2014). Pharmacokinetics and safety of resveratrol derivatives in humans after oral administration of melinjo (*Gnetum gnemon* L.) seed extract powder. J. Agric. Food Chem..

[13] Konno H., Kanai Y., Katagiri M., Watanabe T., Mori A., Ikuta T., Tani H., Fukushima S., Tatefuji T., Shirasawa T. (2013). Melinjo (*Gnetum gnemon* L.) Seed Extract Decreases Serum Uric Acid Levels in Nonobese Japanese Males: A Randomized Controlled Study. Evid.-Based Complement. Altern. Med. eCAM.

[14] Espinoza J.L., Elbadry M.I., Taniwaki M., Harada K., Trung L.Q., Nakagawa N., Takami A., Ishiyama K., Yamauchi T., Takenaka K. (2017). The simultaneous inhibition of the mTOR and MAPK pathways with Gnetin-C induces apoptosis in acute myeloid leukemia. Cancer Lett..

[15] Kolhatkar U., Dholakia K., Sikorska G., Kumar A., Martinez L.A., Levenson A.S. (2018). Abstract 253: Gnetin C as a candidate for targeted chemopreventive and therapeutic measures in prostate cancer. Cancer Res..

[16] Common Terminology Criteria for Adverse Events (CTCAE), v4.0. https://ctep.cancer.gov/protocolDevelopment/electronic_applications/ctc.htm#ctc_40.

[17] Espinoza J.L., Trung L.Q., Inaoka P.T., Yamada K., An D.T., Mizuno S., Nakao S., Takami A. (2017). The Repeated Administration of Resveratrol Has Measurable Effects on Circulating T-Cell Subsets in Humans. Oxid. Med. Cell. Longev..

[18] Trung L.Q., An D.T.T. (2018). Is Resveratrol a Cancer Immunomodulatory Molecule?. Front. Pharmacol..

[19] Blander J.M., Longman R.S., Iliev I.D., Sonnenberg G.F., Artis D. (2017). Regulation of inflammation by microbiota interactions with the host. Nat. Immunol..

[20] Kotecha R., Takami A., Espinoza J.L. (2016). Dietary phytochemicals and cancer chemoprevention: A review of the clinical evidence. Oncotarget.

[21] Falasca M., Casari I., Maffucci T. (2014). Cancer chemoprevention with nuts. J. Natl. Cancer Instit..

[22] Maru G.B., Hudlikar R.R., Kumar G., Gandhi K., Mahimkar M.B. (2016). Understanding the molecular mechanisms of cancer prevention by dietary phytochemicals: From experimental models to clinical trials. World J. Biol. Chem..

[23] Espinoza J.L., Nguyen V.H., Ichimura H., Pham T.T., Nguyen C.H., Pham T.V., Elbadry M.I., Yoshioka K., Tanaka J., Trung L.Q. (2016). A functional polymorphism in the NKG2D gene modulates NK-cell cytotoxicity and is associated with susceptibility to Human Papilloma Virus-related cancers. Sci. Rep..

[24] Espinoza J.L., Takami A., Yoshioka K., Nakata K., Sato T., Kasahara Y., Nakao S. (2012). Human microRNA-1245 down-regulates the NKG2D receptor in natural killer cells and impairs NKG2D-mediated functions. Haematologica.

[25] Espinoza J.L., Minami M. (2018). Sensing Bacterial-Induced DNA Damaging Effects via Natural Killer Group 2 Member D Immune Receptor: From Dysbiosis to Autoimmunity and Carcinogenesis. Front. Immunol..

[26] Bachleda P., Vrzal R., Dvorak Z. (2010). Resveratrol enhances NK cell cytotoxicity: Possible role for aryl hydrocarbon receptor. J. Cell. Physiol..

[27] Li Q., Huyan T., Ye L.J., Li J., Shi J.L., Huang Q.S. (2014). Concentration-dependent biphasic effects of resveratrol on human natural killer cells in vitro. J. Agric. Food Chem..

[28] Lu C.C., Chen J.K. (2010). Resveratrol enhances perforin expression and NK cell cytotoxicity through NKG2D-dependent pathways. J. Cell. Physiol..

[29] Espinoza J.L., Takami A., Trung L.Q., Nakao S. (2013). Ataxia-telangiectasia mutated kinase-mediated upregulation of NKG2D ligands on leukemia cells by resveratrol results in enhanced natural killer cell susceptibility. Cancer Sci..

[30] Quoc Trung L., Espinoza J.L., Takami A., Nakao S. (2013). Resveratrol induces cell cycle arrest and apoptosis in malignant NK cells via JAK2/STAT3 pathway inhibition. PLoS ONE.

[31] Shi Y.W., Wang C.P., Liu L., Liu Y.L., Wang X., Hong Y., Li Z., Kong L.D. (2012). Antihyperuricemic and nephroprotective effects of resveratrol and its analogues in hyperuricemic mice. Mol. Nutr. Food Res..

[32] Chen H., Zheng S., Wang Y., Zhu H., Liu Q., Xue Y., Qiu J., Zou H., Zhu X. (2016). The effect of resveratrol on the recurrent attacks of gouty arthritis. Clin. Rheumatol..

[33] Trautwein E.A., Vermeer M.A., Hiemstra H., Ras R.T. (2018). LDL-Cholesterol Lowering of Plant Sterols and Stanols-Which Factors Influence Their Efficacy?. Nutrients.

[34] Barter P., Gotto A.M., LaRosa J.C., Maroni J., Szarek M., Grundy S.M., Kastelein J.J., Bittner V., Fruchart J.C., Treating to New Targets I. (2007). HDL cholesterol, very low levels of LDL cholesterol, and cardiovascular events. N. Engl. J. Med..

[35] Wilson P.W. (2016). Changing Cholesterol Levels and Coronary Heart Disease Risk. Circulation.

[36] Borges M.C., Lawlor D.A., de Oliveira C., White J., Horta B.L., Barros A.J. (2016). Role of Adiponectin in Coronary Heart Disease Risk: A Mendelian Randomization Study. Circ. Res..

[37] Woodward L., Akoumianakis I., Antoniades C. (2017). Unravelling the adiponectin paradox: Novel roles of adiponectin in the regulation of cardiovascular disease. Br. J. Pharmacol..

[38] Menzaghi C., Trischitta V. (2018). The Adiponectin Paradox for All-Cause and Cardiovascular Mortality. Diabetes.

[39] Ota H., Akishita M., Tani H., Tatefuji T., Ogawa S., Iijima K., Eto M., Shirasawa T., Ouchi Y. (2013). Trans-Resveratrol in Gnetum gnemon protects against oxidative-stress-induced endothelial senescence. J. Nat. Prod..

